# Understanding Learners’ Experiences of Simulated Person Methodology in an Athletic Therapy Program

**DOI:** 10.7759/cureus.7194

**Published:** 2020-03-06

**Authors:** Eva Peisachovich, Celina Da Silva, Natasha May, Michael Boni, Justeena Zaki-Azat, Raya Gurevich-Gal, Loriann Hynes

**Affiliations:** 1 Medical Education and Simulation, York University, Toronto, CAN; 2 Teaching Commons, York University, Toronto, CAN; 3 Health Sciences, York University, Toronto, CAN; 4 Psychology, York University, Toronto, CAN; 5 Medical Education and Simulation, Public Health Service, Beer Sheva, ISR; 6 Kinesiology and Health Sciences, York University, Toronto, CAN

**Keywords:** simulated person, simulation=, pedagogy, athletic therapy

## Abstract

Introduction

Key skills required of today’s students include critical thinking, problem-solving, creativity, innovation, collaboration, and communication. The acquisition of these skills is foundational to success in a variety of professions and contexts. This study complements a larger simulated person methodology (SPM) project that utilizes simulators (individuals who are trained to realistically reproduce scenarios by providing specific information, displaying signs and behaviours, and creating a realistic encounter in a consistent manner) to replicate real workplace issues, thus affording students an opportunity to apply knowledge and practice real-life skills necessary to the workplace. The primary objective of this study is to apply this innovative teaching approach in higher education as a means of developing proficient critical-thinking and interpersonal skills.

Methods

This pilot study uses an exploratory mixed-methods design to explore the experiences of 12 students enrolled in an athletic therapy (AT) certificate program that uses SPM. Our hypothesis is that SPM will have a positive impact on student learning and professional development.

Results

The students responded favourably to the use of SPM. Indeed, 80% “felt challenged and stimulated” and deemed SPM to be a “more effective method” of practicing communication skills than practicing with fellow students. These findings can inform future research and support work towards enhancing this methodology as a pedagogical approach. In tandem, this study and the larger SPM project are poised to provide an effective undergraduate education experience across various faculties at the pilot university. More work is required to align this teaching approach with the AT education program redesign.

## Introduction

Background

Educational systems within Canada are under increasing pressure to use innovative and creative ways to transform education while increasing critical thinking and reasoning skills among learners [1-3]. Further, to succeed in a given practice setting, the following skills are required: professionalism, self-directed learning, leadership, interprofessional communication, and collaboration [4]. This study is a part of an overarching project that is being implemented as a pan-university initiative to build capacity to support the utilization of simulated person methodology (SPM) by educators across disciplines. Within the context of the larger project, the pilot study discussed in this paper was designed to explore the potential of SPM as an educational tool for athletic therapy (AT) students.

Simulation

Simulation has become an integral part of the training of several professions, including aviation (via flight simulators), medicine, and allied health professions. Simulation is a technique designed to replace or amplify real experiences with guided, often immersive, ones that replicate features of the real world in an interactive manner [5]. A large body of literature suggests that simulations have been used reliably in health-professions education to teach and evaluate competencies (i.e., licensing examinations) [6]. Yet SPM is one form of simulation that remains underutilized in many other disciplines, largely due to the costs and the lack of knowledge regarding its application as a methodology in education outside of health professions [7,8]. Research identifies simulation as an approach that supports the synthesis of knowledge and the development of insight [9]. It is therefore essential that teaching institutions take a participatory and collaborative approach in the application, development, and use of this methodology for training novice professionals. 

Simulated person methodology

SPM is one simulation-based learning approach that is widely cited in the literature. It features the use of a simulated person (SP) [9]. SPM is a learner-centred, experiential-education approach that bridges theory and practice by providing students with practical experiences and the ability to reflect on these experiences using their theoretical knowledge [9]. Further, it provides an opportunity for active and interactive learning in a realistic simulated environment [10]. It is related to situated-learning theory, which posits that learning often happens naturally and is embedded within the context, culture, and activity in which it occurs [10]. Other studies conducted within nursing and medical schools, not only in North America but also in Australia, report similar findings regarding the benefits of simulation and SP interactions [11].

An SP is an individual who is trained to portray a role for learning or assessment purposes [12]. SPs are trained to realistically reproduce learning scenarios by providing specific information, displaying signs and behaviours, and creating a realistic encounter in a consistent manner [12]. Studies conducted thus far reveal similar results, indicating that SPs contribute positively to learning and knowledge or skill acquisition and retention for students in both the short and long term [13,14]. In follow-up debriefings, SPs are trained to provide learners with feedback on professional manners, attitudes, and interpersonal skills, thus promoting individualized, in contrast to traditional standardized, learning. Moreover, feedback is immediate and from the “person’s” point of view. The amount of standardization of the SP varies according to the simulation; in formative learning sessions, the aim is toward individualized learning; in standardized, summative, or high-stakes assessments, the aim is to ensure validity and reliability. Our focus in this study is both on formative and summative assessments to provide students with exposure to both individualized and standardized SP interactions.

SPM provides learners both a safe environment in which they can make mistakes without causing harm (as may occur in real-life situations) and an opportunity to reflect on and learn from these mistakes. This provides learners who are exposed to SPM with an opportunity to make meaning out of interactions and to develop communication and interviewing skills in a safe environment. Despite the promise of SPM, limited research has been conducted on its application, its impact on student learning, or its success in professional development outside fields such as medicine and nursing [13,14]. Moreover, limited knowledge exists on the effectiveness of SP encounters in professional programs other than healthcare professions, such as law, business, social work, librarianship, and education [15]. SPM may benefit these fields as it could, for example, allow law-enforcement students to practice de-escalating and negotiating skills, law students to hone interpersonal skills needed for successful client relations, or future educators to develop the sensitivity needed to talk with parents regarding their child’s performance or behaviour. Further, clinical-placement shortages are prevalent in various areas of education, which makes this simulation method an opportunity for learners to practice and access environments that do not match the textbook portrayal [16]. The integration of SPM into Ontario’s changing healthcare system could be an effective way to address these shortages.

Simulation and athletic therapy

Although simulation has been used to a certain extent in the specialized orthopaedic rehabilitation profession of AT as a teaching and learning strategy, it has not been extensively researched; nor has the use of SPs been researched as an evaluation strategy. Although there have been studies regarding the effective use of high-fidelity simulators (such as manikins), the application of SPs has not been extensively studied [17,18]. High fidelity is defined as “how accurately reality is represented”, but it is important to consider that fidelity is perceived at emotional, conceptual, and physical levels [19]. Hence, people may perceive or experience different degrees of realism when immersed in the same simulation; realism, therefore, becomes the property of the learner’s perception and not of the simulation.

Themes emerging in studies employing SPs in AT are similar to those seen in other SPM studies; these include increasing confidence and experience with real-time patients, developing interpersonal skills, and having an effective method for evaluation and constructive feedback [18,20-22]. AT students found the learning experience valuable, which allowed them to develop clinical skills such as interacting with patients and taking their health histories and to reflect on their provision of patient care [20,21). Thus, engaging students in active learning through various forms of simulation and role-play has been shown to be beneficial for the students in terms of increased self-efficacy, knowledge acquisition, and retention and fostering of clinical skills [23].

An AT education curriculum includes in-class competency courses, such as prevention and evaluation techniques that are correlated with clinical experience; however, employers are finding that new graduates often lack assertiveness, communication, and initiative skills [23]. Currently, AT programs use a variety of assessment tools to evaluate clinical competency, including checklists, proficiency-rating scales, visual scales, and open-ended questions, but there is an increasing need to provide students with sufficient experiential learning to enhance interpersonal, communication, and leadership skills [24].

A study conducted in the United States educational context has found that students in AT training programs are being evaluated in an actual clinical setting where they interact with real patients less than 50% of the time [18,20,21]. Bringing SPM into the classroom would afford both exposure prior to practice and interaction with real persons, thus providing students a safe space to make errors and reflect in action and on action. Hence, the research question is: what are the experiences of AT students through using SPM within the classroom and its exposure prior to practice through interaction with SPs?

## Materials and methods

An exploratory mixed-method design was employed both for this pilot study and for the larger, complementary project being conducted by the authors. This approach was chosen for the pilot study under discussion as it allows investigators to garner invaluable insight into an experience or phenomenon by leveraging the strengths of both quantitative and qualitative methods [25]. This pilot study was approved by the review board of the York university’s ethics committee with the certificate no: e2016 - 362.

Participants

The AT program at York University consists of a cohort of 12 students: 12 undergraduate students (four male and eight female participants with an average age of 24), which is representative of the overall student body in the program, who were enrolled in the kinesiology undergraduate degree and were in the final semester of the 3-year AT certificate program at a Canadian university. The students were informed about the pilot study at the beginning of the term by the members of our research team at which time 100% of the study participants self-selected to participate, which was followed by signing a consent form. The final-year Advanced Athletic Therapy Assessment and Rehabilitation course consisted of 12 students, and all 12 were invited to participate through an announcement that there would be a formative and a summative activity in which students could interact with SPs. Although students had experienced other modes of simulation, including high-technology manikins and role-play, they had not interacted with SPs prior to this study, which our research team found out after questioning the participants once they agreed to participate.

Simulated persons

The SPs worked closely with SP trainers and the faculty member to create roles for use in the classroom. Four training sessions were conducted: two for the formative activity and two for the summative. Six SPs, one SP trainer, and three faculty members teaching the course attended each training session. The first training session focused on reviewing the scenario template, ensuring that the SPs were comfortable with portraying certain movements and that the information including scenarios, role objectives, role descriptions, and SP prompts was clear. The faculty members then rotated to interact with SPs as learners. The roles were practiced with faculty members in the position of the learner experiencing simulation. The SP trainer guided this experiential component by highlighting approaches appropriate with different types of learners and discussing the facilitation skills and the level of detail necessary to support SPs in the simulation. This partnership allowed these roles to be refined to meet the curriculum and learning objectives. SPs were given an opportunity to apply their knowledge of SPM by portraying patients, family members, and team members in simulated case scenarios designed to replicate situations students are likely to encounter in their future workplaces. The participants were introduced to the concept of simulation and were given an overview of the scenario and objectives prior to the first interaction.

Using the SPM feedback model, the SPs provided students with feedback on their communication, professionalism, and interpersonal skills after every interaction [26]. The SPM feedback model, which the SPs in the study practiced during their training sessions, provides SPs with a structured way to offer feedback to learners:

· Begin with the person receiving the feedback. Ask for their experience; it helps you to know what was going on for them.

· Remember that we can never know what somebody else is thinking, feeling, or intending.

· Ask rather than tell.

· Consider what things about the learner or interaction could interfere with someone’s ability to receive feedback.

The SPs are trained to provide effective feedback by being specific, not hypothetical; avoiding would and should; avoiding assumption and judgment; relating feedback to learning objectives; basing feedback on what was actually said or done and on the response to it. In SPM, the feedback is given from the SP’s experience, not from the character’s. Feedback is provided by using this structure: “When you said (or did) ______________I felt, thought, or experienced ___________.” For example, an SP might say, “When you started explaining why you called me in and you looked at the file the whole time, I thought maybe I had done something wrong. I was scared and felt disrespected and unimportant.”

Procedure

After announcing the study to the students in the course, they were informed of the voluntary nature of the study and were assured that there would be no consequences resulting from their declining to participate. If they did not want to participate, an alternate evaluation option was available. An informed consent form explaining this was signed by each of the students who chose to participate.

The first simulation was used as a formative evaluation halfway through the course, while the second simulation was used as a summative evaluation four months later, at the end of the course. Using both these evaluation approaches enriched the extent to which SPM can be used to enhance student competence and professional development.

Two student-SP interactions were implemented to provide one formative session, which included history-taking and orthopaedic joint assessment, and one summative, full uninterrupted assessment as well as a single-stage rehabilitation program design and implementation.

Each simulation consisted of an interaction (which lasted between 15-20 minutes) between the SP and the student, followed by approximately 30 minutes of feedback provided by the SP. Following that, a 30-minute debrief and evaluation was facilitated by an instructor. Each simulation scenario represented competencies and psychosocial issues students may encounter in practice as athletic therapists. Data were collected at two interval points: a follow-up survey was distributed after the first simulation debrief, and a focus-group interview was conducted following the second simulation debrief. Both the survey and the focus-group interview provided insight into the students’ experience with the SP encounters.

Formative SPM Simulation

A formative assessment afforded an opportunity for participants to practice and demonstrate their learning; the main goal of this assessment was to help participants improve through feedback. The formative assessment occurred halfway through the course and involved meeting course objectives through an SP encounter. The 12 participants were divided into six pairs, and each pair was assigned one SP; one participant had to collect health history and the others had to conduct a physical exam aligned with the SP role in the scenario objectives.

Each participant had the opportunity to either interview the SP regarding general and injury history or to conduct a basic orthopaedic physical exam of the shoulder. To measure students’ experience of the effectiveness of the SP encounter, a 24-item follow-up survey was distributed to the participants once the formative evaluation was completed.

Summative SPM Simulation

The second SP activity in our study occurred at the end of the course and was summative in nature. In contrast to formative evaluations, summative assessments require students to demonstrate their learning with the purpose of summarizing their achievement; such evaluations often affect decisions regarding success or failure. The simulation entailed a full history and orthopaedic examination pertaining to an ankle injury; additionally, participants directed SPs through a single phase of rehabilitation, to be conducted post-assessment. To measure students’ experience of the effectiveness of the SP encounter, the evaluation was followed by a focus-group interview conducted prior to the participants receiving their summative evaluation results.

Measures

Follow-up Survey 

A follow-up survey (Table 1) was developed for this study using content-expert feedback; it was adapted from a questionnaire from a validated survey research project used in a recent publication by one of our co-authors and was used with permission.

**Table 1:** Follow-up survey N/A: not available; SP: simulated person

Learning need	Simulated environment						Importance of learning need		
	Strongly agree	Agree	Disagree	Strongly disagree	Not applicable		Agree	Disagree	Not applicable
C = COMMUNICATION									
C1. The simulation provided an opportunity for effective communication with the client/patient	4	3	2	1	N/A		2	1	N/A
C2. Practicing with the SP in the classroom is an effective method for developing communication/interviewing skills	4	3	2	1	N/A		2	1	N/A
C3. Practicing communication skills with the SP is a more effective method than practicing with classmates	4	3	2	1	N/A		2	1	N/A
A = AUTHENTICITY									
A4. The simulation provided authentic interaction with the client/patient	4	3	2	1	N/A		2	1	N/A
A5. I was able to perform an appropriate assessment	4	3	2	1	N/A		2	1	N/A
A6. I was able to identify the client/patient’s problem/condition	4	3	2	1	N/A		2	1	N/A
A7. I was able to prioritize my assessment components	4	3	2	1	N/A		2	1	N/A
A8. The simulation allowed me to recognize the significance of client/patient’s responses to history questions	4	3	2	1	N/A		2	1	N/A
A9. The simulation allowed me to recognize the significance of client/patient’s responses to the physical assessment	4	3	2	1	N/A		2	1	N/A
A10. The simulation provided the realism/authenticity of client/patient interaction	4	3	2	1	N/A		2	1	N/A
A11. The interaction with the SP has allowed for implementing decision-making skills	4	3	2	1	N/A		2	1	N/A
A12. The simulation allowed for the integration of assessment results	4	3	2	1	N/A		2	1	N/A
A13. I felt challenged and stimulated	4	3	2	1	N/A		2	1	N/A
A14. I felt confident in my abilities	4	3	2	1	N/A		2	1	N/A
A15. The simulation provided me with the opportunity to know what to do	4	3	2	1	N/A		2	1	N/A
A16. The simulation improved my critical thinking skills	4	3	2	1	N/A		2	1	N/A
A17. The simulation contributed to my knowledge of assessment procedures	4	3	2	1	N/A		2	1	N/A
A18. Engaging with SP prepared me for working with real clients in the workplace or practice	4	3	2	1	N/A		2	1	N/A
F = FEEDBACK									
F19. The simulation experience provided immediate feedback	4	3	2	1	N/A		2	1	N/A
F20. The feedback received from the SP was effective to my learning	4	3	2	1	N/A		2	1	N/A
F21. Feedback about my interactions with the SP was provided in a timely manner	4	3	2	1	N/A		2	1	N/A
F22. Feedback provided by the SP was helpful for my learning	4	3	2	1	N/A		2	1	N/A
F23. The feedback from the SP has allowed for self-reflection	4	3	2	1	N/A		2	1	N/A
F24. The feedback from the SP has allowed me to make decisions in a more effective manner	4	3	2	1	N/A		2	1	N/A

The use of a 4-point scale with the option of N/A (not available) off-scale is a strategy recommended by researchers to avoid the misuse of midpoints as a “dumping ground” when they are responding to survey items that are unfamiliar to them [27]. The questions probed three learning domains: communication, authenticity, and feedback. Each item across the three domains was measured on a 4-point Likert scale to provide an indication of how well (strongly agree to strongly disagree) the specified learning point was made. The more they agreed that the learning point was made, the higher their score on the item, as well as on the learning domain. The follow-up survey items probed SP performance; student-SP interaction; relevance to students’ professional education; SP efficacy as a strategy for learning; and the authenticity, relevance, timing, and perceived benefit of feedback from SP interactions. Participants were also given an opportunity to agree or disagree on the importance of the “learning need” item.

A paper survey was distributed with no online distribution option. The survey was analyzed using Statistical Package for the Social Science (SPSS) software (IBM, Armonk, NY). Names of the participants were not included on the survey sheets; the surveys were number coded from one to 12 and were not shared beyond the research project team.

Focus Group Interview

The focus-group interview questions were asked after the second simulation and were not informed by the follow-up survey results (Table 2).

**Table 2:** Focus group interview questions SP: simulated person

Focus group interview questions
What have you learned from having the experience of a scenario enacted in this way? What did you learn?
What do you think are the benefits of learning in this way?
What are the challenges of learning in this way? Have you encountered or witnessed challenges during the interaction with the SP? Describe your experience
What were the strengths of this methodology? The SP?
Did you benefit from the feedback?
What did you take away from this experience?
Did you learn anything about yourself from this experience with the SP?
How do you describe this experience’s relevance to your practice placements?
Rate the authenticity of the scenario of the SP, as best as you can. Number one, the scenario felt completely authentic. Number two, the scenario was very authentic. Number three, the scenario was somewhat authentic. Number four, the scenario was not very authentic and number five, the scenario was completely inauthentic
Provide the rationale for the rating
Is there anything you would like to share about your experience that we didn’t ask you about?

Focus groups offer multiple perspectives through group interaction and are especially useful for exploring people’s experiences, what they think, and why [25]. The study’s focus group provided relevant, detailed information about the group’s perceptions, feelings, and opinions related to the SP experience; further, it provided the facilitator an opportunity to seek clarification through focus-group questions. Data collected through this interview offered a broad range of information about the events and experiences of students, thus providing first-hand knowledge of their experiences with SPM. The focus-group feedback was transcribed and coded to identify themes.

Data analysis

Follow-up Survey

Due to the small sample size used in this pilot study, our approach was to examine the frequencies of the participant responses as an indication of how well the specified learning point was made to the participants. Nonparametric statistics were also appropriate to explore relationships between the items using Kendall’s tau-b correlation coefficient analysis. Since a regular chi-square does not allow the analyst to consider the (quasi-)ordered nature of the categorization, we decided to use Kendall’s tau-b-a nonparametric measure of the strength and direction of association that exists between two variables measured on an ordinal scale. The benefits of Kendall’s tau-b include its appropriateness for a small sample and its provision of data that is not continuous.

Focus Group Interview

Thematic analysis was used to analyze focus-group data, which was transcribed verbatim [25]. The analysis process included (a) familiarization: reading through the data and recording insights and reflections; (b) coding: “selectively attach[ing] meaningful tags to words, phrases, events, situations, and so forth, naming what is potentially important about them, and distinguishing them from the rest of the data” [28]; (c) sorting data to identify similar phrases, patterns, themes, sequences, and other important features; (d) looking for commonalities and differences among the data and extracting them for consideration and analysis; (e) gradually deciding on a small group of generalizations that hold true for the data; and (f) examining these generalizations in light of existing knowledge [28]. To ensure trustworthiness in all qualitative analyses, research-team members conducted the initial coding. Discrepancies in coding were discussed among research-team members to reach an agreement and form a unified coding scheme that was used to (re)code all data. The entire team was involved in categorizing or clustering themes, discussing interpretations, and agreeing on selected excerpts from participant data. We have attended to the authenticity of interpretation and audit trails to show a logical progression from raw data to themes or abstractions.

## Results

Follow-up survey

**Figure 1:**
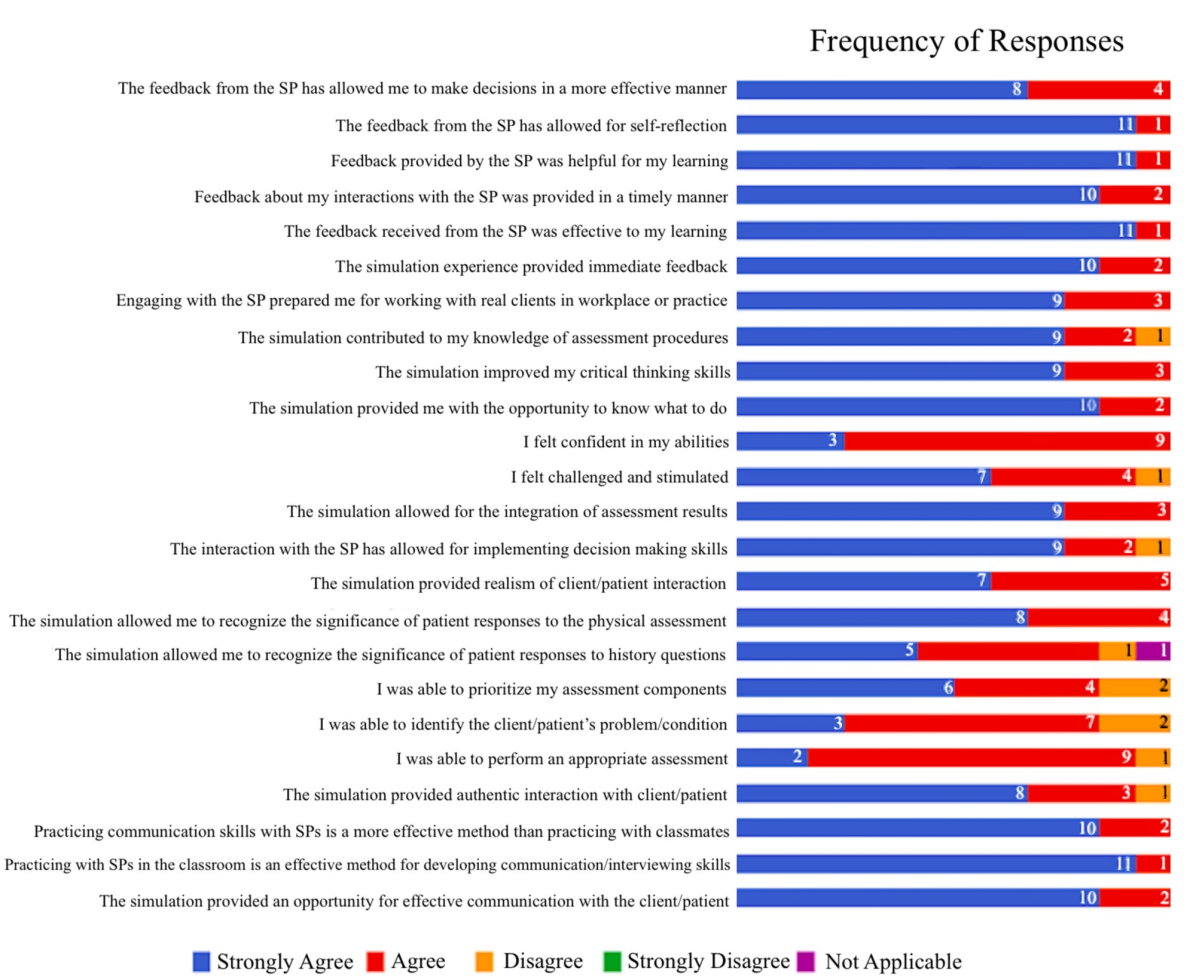
A display of the frequency of responses to each item of the follow-up survey SP: simulated person

The findings support the notion that the opportunity to practice the development of communication and interviewing skills with SPs in the classroom, as was done in these formative and summative learning experiences, directly increases the participants’ feelings of being challenged and stimulated in the learning environment. The majority of participants responded that they strongly agreed or agreed that they had learned through the different aspects of the simulation environment. A display of the frequency of responses to each item of the follow-up survey is shown in Figure 1. Not one single participant strongly disagreed with any item.

**Figure 2:**
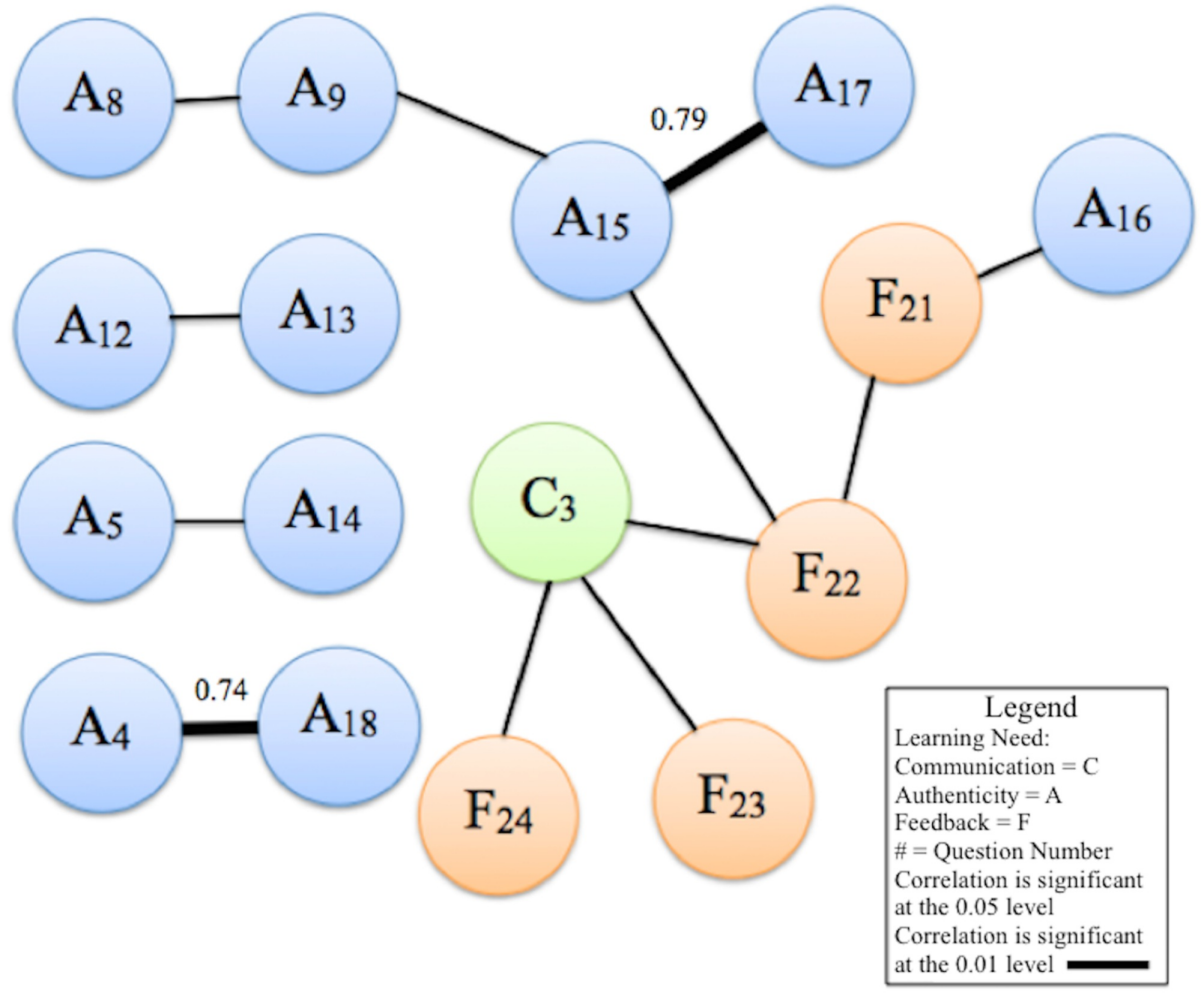
Constellation diagram of correlations among follow-up survey responses

Four networks of correlations were found, and the results of the analysis were plotted as a constellation diagram of correlations (Figure 2).

Correlation coefficients indicating the strength of the relationship between items are labeled for correlations significant at the alpha .01 level. The constellation diagram displays the data on two-dimensional x-y plots with nodes and links, which are used to describe the correlation among component nodes. The constellation diagram is useful in visualizing the associations, as this paper is largely exploratory and has a small sample size. Due to multiple Kendall’s tau correlations run with each combination, we are only reporting the correlations that are statistically significant (p = <.05). These correlations indicate a need for greater focus in the areas captured by the items. There are other strong correlations that are not statistically significant and, thus, have been excluded for the purposes of this discussion.

The first correlation (b = .71, p = .015) was identified between the items “the simulation allowed for the integration of assessment results” (A12) and “I felt challenged and stimulated” (A13). A second direct correlation occurs between the items “I was able to perform an appropriate assessment” (A5) and “I felt confident in my abilities” (A14) (b= .68, p = .021). Similarly, a third pair of items, “the simulation provided authentic interaction with the client/patient” (A4) and “engaging with the SP prepared me for working with real clients in workplace or practice” (A18), was correlated at a highly significant level (b = 0.74, p = .009).

The fourth and largest network of this correlation bond demonstrates a complex interconnection of all learning needs, with communication as the integral component. The item “practicing communication skills with SPs is a more effective method than practicing with classmates” (C3) acts as a crucial bond through which all learning domains are related (Figure 2). The effectiveness of communicating with SPs, rather than with classmates during role-play, is strongly seen in relation to the responses given: the specific items that support SPM’s effectiveness in developing communication are its helpfulness in learning (F22), allowance for self-reflection (F23), and efficacy in facilitating decision making (F24). The aforementioned items are directly related to perceptions of the efficacy of communication in simulation over role-play with classmates and are significant correlations. Through the item of helpful feedback (F22), communication (C3) is connected to authenticity and realism. This speaks to the strong implications of communication in this methodological approach.

Focus group interview

Five themes emerged. Other findings were not significant but included one challenge: two participants commented on the fact that the SPs could not answer all of their questions, as opposed to the role-play where their “colleagues” would provide the answers. One participant elaborated, “We had to dig deeper into the issue to figure out what the patient was experiencing.” This “challenge” could alternatively be considered as a strength of the methodology. 

The five significant themes that emerged from this process are the importance of professionalism, experiential variance from the role-play, formative soft-skill development, the value of feedback, and relationship building.

Importance of Professionalism

The reference to the skills and practices that contributed to professionalism was recurrent. The interaction with the SP, as someone unknown to the participants, contributed to the application of professional practices such as the introduction of self, role, and purpose of interaction. Participants shared the following thoughts:

“I learned that it is important to introduce yourself (learner introduced himself to SP or patient). . . . We need to introduce ourselves to make them feel comfortable and that’s kind of a priority and also discussing things in less technical terms so they can better understand it. I think that’s a good thing that we are not used to at all.”

“You definitely learn how to be more professional when working with simulated patients. It is kind of like that first impression was very important with that. I think that is very related to the clinical setting, like your first impression is everything.”

Experiential Variance from Role-play

Participants highlighted the differences between learning with SPM and that with role-play, which is frequently used in their program. They noted that working with the SP required consideration of components of the patient experience that were assumed when practicing with peers:

“When we are working on each other we assume what we are going to do, in terms of resisted testing or doing a specific test, so the person with a classmate are already pre-anticipating the movement or the exercise that we are going to do, so using these simulated patients forces us to go into full explanation on how we have to do things because they don’t know how we are going to do them.”

Another participant noted the difference in experience and interaction between simulation with an SP and role-play with peers or others already known to them: 

“Because they [fellow students] know us on a regular basis including, instructors or other people in our class, the [simulated] patient we saw a couple of weeks ago even today, for the most part, we have no idea who they [are], they have no idea who we are which makes the interaction real.”

Formative Soft-skill Development

Soft skills, or interpersonal skill development, were significant to learners. It was indicated that clinical knowledge was easy to apply, as they know what to do; however, the value of the interaction was in translating this in a relatable way to the client. Participants shared the following thoughts:

“I think what this helped us with, well me, in particular, is those fine skills, the soft skills our professors are talking about.”

“We don’t ask [about] comfort level, because we assume they will tell us if they are not comfortable. It is all of those soft sorts of skills, is something that I got.”

Value of Feedback

SPM includes a framework for feedback from the perspective of the SP. The participants highlighted the value and benefit of this feedback in their practice:

“I think that more feedback from your [the facilitator or instructor] side of things, from the patient [the SP] themselves for me it was very helpful.” 

“They were able to comment on something that our instructors or our evaluators can’t see, which is how comfortable they feel.”

Relationship Building

Building a relationship was a key theme that arose out of the participants’ interactions with an SP. This critical aspect of practice was salient to effective engagement with their clients. Participants shared the following thoughts:

“You can be a great clinician, but if you are not making your patient feel comfortable, anything you do won’t change their mind. In terms of what you are able or capable of doing, so I feel like that is huge.”

“And I think especially that initial aspect of building that relationship and, again, like she said making them feel comfortable and trusting you.”

## Discussion

One consistent challenge among professional healthcare education programs is providing students with realistic environments or situations that support their transition to the workforce. The soft skills required to make strong and trustworthy professionals are easily overlooked in a classroom setting and replaced with theoretical teachings. The “art” of human interaction can be developed in professional-placement environments, but these opportunities can be challenging to secure due to availability (due to lack of proximity to educational institutions and large class sizes) and the need for supervisors with a capacity to oversee student interactions and provide feedback and evaluation. As a result, laboratory-style courses are designed to provide learners with opportunities to practice real-life application of skills in an environment designed to prevent harm if errors in judgement are made. While role-play has been used extensively in the AT programs, the evidence from this study suggests the potential of SPM as a means of improving the human-interaction skillset of the student, particularly as it relates to appropriate communication and professionalism, and underscores the need for larger surveys to confirm this pilot study’s promising results.

Using SPs is an alternative way to allow students to gain the necessary clinical skills that they would otherwise gain in their clinical placements [29]. SPM provides students with the opportunity to be participants in their own learning as opposed to performing correct and incorrect actions. With SPM, students can construct meaning about the world. The study results demonstrate that students found the experience valuable, as they felt it provided them with the clinical reasoning and communication skills necessary to grow professionally by affording them an opportunity to develop cognitive, psychomotor, and teamwork skills. The development of these skills suggests that this type of teaching methodology can be used in a wide variety of programs; it can, for example, be used to prepare for such things as interviews, conflict management, and crisis intervention. We suspect that it is not limited to healthcare professions.

Further, the findings of this study show that SPM provides learners with opportunities to be participants of their learning and solidifies the notion that this form of learning allows learners to construct meaning about the world and their role in it, as they develop professional competence; additionally, it provides an opportunity to reflect in action and on action. Further, the results illustrate that communication, engagement, and feedback are imperative in order to develop professionally. One participant indicated that the simulation provided him with an opportunity to practice asking open- and close-ended questions:

“I found that for me specifically . . . what I learned . . . there was a big difference between open- and close-ended questions . . . [when] the patient, doesn’t give me the information I was looking for . . . so I had to dig even deeper and I thought that by asking more questions I’d get that information which made me realize the importance of how I ask questions.”

Moreover, another participant corroborated that the feedback provided by the SP provided an opportunity to self-reflect and to take this feedback into the practice milieu:

“We don’t get feedback from real patients, and you get feedback from these [SPs] patients, which allows you take those lessons into your practice.”

 The participants indicated that the methodology led them to an awareness about their approach, such as the need to avoid applying too much physical pressure during the exams or the criticality of introducing oneself; in other words, the methodology allowed them to improve their soft skills. The feedback from the SPs also gave them a chance to work on their skills and facilitated critical thinking and self-reflection, thus preparing them to practice in complex and dynamic workplace environments.

Limitations

Although the sample size was small, it was appropriate for preliminary research and appropriate in the context of this study. The small number of students participating in this study, and their enrollment in the AT program, limit the researcher’s ability to generalize the ﬁndings to other populations. It should also be noted that research was conducted with the sample performing as a group, as opposed to individually, which could challenge the validity of the ﬁndings. Although the ﬁndings contribute to the literature on the use of SPM, a larger sample size may have generated more data. In future studies with a similar focus, a larger sample size may lead to diverse perceptions and more extensive ﬁndings. As the total population of senior AT students in all of the country amount to about 250, sample size calculations show that a sample size of at least 50 participants would be deemed a sufficiently large sample to be able to draw conclusions from with 90% confidence. Since the current York University AT program houses on average 20 students, further studies would ideally be a collaboration between institutions to recruit such a sample.

Our pilot study suggests that the benefits of SPM may extend beyond healthcare education to a myriad of other disciplines, including AT. AT programs may benefit on multiple levels by incorporating SPM into their learning environments. More research is needed in the area of AT and the use of SPs, and this study contributes to the limited body of knowledge in this area.

Although the study shows that the application of SPM provides a positive correlation between variables and a high level of student satisfaction, perceived barriers to implementation exist, mostly due to its high cost [8]. Other barriers, such as laboratory space, time, and financial support, have also constrained AT programs from fully incorporating SPM at this time [18]. To address the issue of costs, the application of simulation can be incorporated into the curriculum as an elective, allowing students to take the course for credit that teaches them about SPM as an experiential education method. A model for the effective application of SPM within higher education has been posited, and it allows students to enroll in such a course which includes a practicum element that provides an opportunity to practice as an SP; however, this model has not been piloted yet.

Although this study answers a number of questions about AT students’ experience with SPM, many questions still remain: how does this experience transfer to the clinical arena? What associated patient-care outcomes are aligned with implementing this methodology? These remaining questions may be better addressed by other researchers from different cultural or professional backgrounds, such as practice leaders, academics and policymakers, and those from different conceptual frameworks, as they may bring a different perspective to the meaning of clinical reasoning and its integration in the transition to practice.

Recommendations and next steps

The National Athletic Trainers’ Association Executive Committee for Education promotes using “innovative teaching and learning methodologies” to better prepare students and improve current classroom and clinical settings [23].

The Athletic Therapy Certificate Program (ATCP) in which this study was conducted is currently undergoing curricular restructuring. The recently approved structure includes a student-centred, inquiry-based model; the results of this study support the integration of SPM into this new model of program delivery. SPM will be integrated throughout the three years of the ATCP using discrete elements of “patient” assessment and rehabilitation to afford students an opportunity to learn basic communication skills in the first year. In the second and third years, the methodology will expand to encompass greater tasks, length of scenarios, and evaluative components with SPs, providing valuable feedback for students to reflect upon their own abilities and values in an effort to construct plans for continual improvement.

Given the small sample size of the study, we recommend examining the SP-student interaction approach with a larger sample size and with participants enrolled both in the ATCP and in other disciplines outside of health. Furthering the construction of a valid and reliable scale from the 24 existing items, the use of Cronbach’s alpha will allow for a better understanding of the relationship between the items.

Due to the high correlations found, the results of this study persuaded the university that hosted this pilot study to support the integration of SPM into the new model for its ATCP. Further, this study supports the merits of SPM, even given the associated costs of such a practice.

## Conclusions

Overall, the findings of this pilot study make a useful contribution to the growing literature on the use of simulation as an innovative approach to active learning in higher education. While the findings, including increased communication skills and professionalism, underscore the promise of this approach, it is important to consider that the SPM approach is costly and requires diligent preparation and delivery. When making a change in the curriculum, success is often dependent on the manner in which the change is implemented. While the methodology required substantial time and resource commitment up front, future course modifications are expected to take less time to create and implement. Although studies have been conducted regarding SPM application in other professional programs, this is one of the few studies to examine using SPs as a form of high-fidelity simulation in AT certificate courses, in which students are engaged through different types of active learning in the classroom.
